# Rapid decrease of malaria morbidity following the introduction of community-based monitoring in a rural area of central Vietnam

**DOI:** 10.1186/1475-2875-8-3

**Published:** 2009-01-05

**Authors:** Ngo Duc Thang, Annette Erhart, Le Xuan Hung, Le Khanh Thuan, Nguyen Xuan Xa, Nguyen Ngoc Thanh, Pham Van Ky, Marc Coosemans, Nico Speybroeck, Umberto D'Alessandro

**Affiliations:** 1 National Institute of Malariology, Parasitology and Entomology, Luong The Vinh street 245, BC 10200 Tu Liem district, Hanoi, Vietnam; 2 Prince Leopold Institute of Tropical Medicine, Nationalestraat 155, 2000 Antwerp, Belgium; 3 Provincial Centre for Malariology, Parasitology and Entomology, 156 Ngo Gia Tu, Phan Rang, Ninh Thuan, Vietnam; 4 Ecole de santé publique, Université Catholique de Louvain, Clos Chapelle-aux-Champs, 1200 Bruxelles, Belgium

## Abstract

**Background:**

Despite a successful control programme, malaria has not completely disappeared in Vietnam; it remains endemic in remote areas of central Vietnam, where standard control activities seem to be less effective. The evolution of malaria prevalence and incidence over two and half years in a rural area of central Vietnam, after the introduction of community-based monitoring of malaria cases, is presented.

**Methods:**

After a complete census, six cross-sectional surveys and passive detection of malaria cases (by village and commune health workers using rapid diagnostic tests) were carried out between March 2004 and December 2006 in Ninh-Thuan province, in a population of about 10,000 individuals. The prevalence of malaria infection and the incidence of clinical cases were estimated.

**Results:**

Malaria prevalence significantly decreased from 13.6% (281/2,068) in December 2004 to 4.0% (80/2,019) in December 2006. *Plasmodium falciparum *and *Plasmodium vivax *were the most common infections with few *Plasmodium malariae *mono-infections and some mixed infections. During the study period, malaria incidence decreased by more than 50%, from 25.7/1,000 population at risk in the second half of 2004 to 12.3/1,000 in the second half of 2006. The incidence showed seasonal variations, with a yearly peak between June and December, except in 2006 when the peak observed in the previous years did not occur.

**Conclusion:**

Over a 2.5-year follow-up period, malaria prevalence and incidence decreased by more than 70% and 50%, respectively. Possibly, this could be attributed to the setting up of a passive case detection system based on village health workers, indicating that a major impact on the malaria burden can be obtained whenever prompt diagnosis and adequate treatment are available.

## Background

In 1991, Vietnam experienced a devastating malaria epidemic that caused more than one million cases and 4,646 deaths. The same year the National Malaria Control Programme (NMCP) was launched and since then, thanks to the political commitment, the fast economic growth and strong international support, the burden of malaria has reduced dramatically [1,2]. Indeed, in 2006 only 91,635 malaria cases and 43 malaria deaths were reported nationwide, a 95% and 99% decrease respectively compared to the 1991 figures [3]. The national insecticide-treated bed net (ITN) campaign, supported by an intensive media campaign on the importance of malaria and ITN use, and the widespread use of artemisin derivatives for treatment, were at the basis of such a success. In addition, indoor-residual spraying (IRS) was employed in epidemic prone-area or where ITN coverage was low [1]. The recent development of Long-Lasting Insecticide Nets (LLINs) should overcome the problems of low re-treatment rates, washing and variation in insecticide dosing, possibly improving their effectiveness [4]; however this new tool is not yet available in Vietnam. Despite past successes, malaria has not completely disappeared in Vietnam; it is confined to some remote areas, mainly inhabited by ethnic minorities. Besides the burden on the local populations, malaria affects also migrant workers from non-endemic areas, with the potential of spreading where transmission has virtually stopped. Therefore, though geographically limited, the control of malaria in these areas is extremely important for the whole Vietnam and possibly for its neighbouring countries. Currently, about half of all malaria cases and 80% of severe cases and malaria-related deaths occur in the central highlands [5-7], where the main vector is *Anophele dirus s.s*., a highly anthropophilic sylvatic species, whose exophagy and exophily as well as early biting habits limit the impact of interventions such as IRS or ITN [8,9] Indeed, in central Vietnam, forest activity has been identified as a strong risk factor for malaria infection [10-12]. It is, therefore, important to know the epidemiology of malaria in these areas to understand its dynamics. A longitudinal malaria surveillance system consisting of bi-annual cross-sectional surveys and passive case detection of malaria cases at community level, through an extensive network of trained hamlet health workers, was set up in Ninh Thuan Province (centre-south Vietnam) as a component of a larger study aiming at evaluating the effectiveness of long-lasting insecticidal hammocks. This paper reports the evolution of malaria prevalence and incidence in this population during a 2.5-year surveillance period by community-based monitoring of malaria cases.

## Methods

### Study site

The study was carried out in two neighbouring districts, Bac Ai (eight communes and 25 villages) and Ninh Son (two communes and five villages), situated in the hilly and forested part of Ninh Thuan province (southern coast of central Vietnam). The population is mainly represented by the Raglai ethnic group, whose members practise subsistence farming (maize, cashew, rice, beans and manioc), cultivate cash crops such as coffee and cotton, and exploit forest products (bamboo, resin, hunting).

The climate is a combination of tropical monsoon and dry and windy weather. The dry season occurs between January and April, with the coldest period in January and February, and the rainy season between May and December. The mean rainfall is 725 mm/year, with the mean temperature ranging between 25°C and 30°C and the humidity between 70% and 80% (Ninh Thuan provincial climatic data, from 2000 to 2006). Malaria transmission is perennial with two peaks, one in June and the other in October. Twenty two different Anopheles species have been identified: the two main malaria vectors are *An. dirus s.s*. and *Anopheles minimus A*, while secondary vectors such as *Anopheles maculatus *or *Anopheles jeyporiensis *probably play a non-negligible role in the local malaria transmission (Van Bortel & Coosemans, personal communication).

### Census

A full census of the study population was done in March 2004: information on age, sex, socio-economic status, forest activity, bed net availability and previous vector control measures was collected. [12]. Each individual living in the study area was attributed a unique identifier code (including village, house and family member codes) which was used in all study activities (census update, surveys, PCD). The census file was routinely updated as births, deaths and migrations were collected by village health workers (VHW) and reported monthly to the malaria provincial station where the electronic census file was managed. The study area was divided into clusters of 1 to 3 neighbouring villages in order to reach a total of about 1,000 people per cluster. Size and number of clusters were determined according to the cluster design requirements and the expected effect of the intervention [12].

### Cross-sectional surveys

Each year, between April 2004 and November 2006, two cross-sectional surveys, one at the beginning (April, May) and one at the end of the transmission season (November, December), were carried out. A random sample of 160 individuals aged 10–60 years per cluster was selected for the first survey in April 2004 [12]; and was subsequently increased from the 2^nd ^survey onwards by 60 additional and randomly selected children aged 2–9 years, lately identified as a high risk age group. An individual, pre-coded standardised questionnaire was administered on previous malaria symptoms and anti-malarial treatment taken, and a clinical examination, body temperature and spleen size, was carried out. A blood sample (finger prick) for thick and thin blood film was collected. Suspected malaria cases were presumptively treated either with chloroquine (25 mg/kg in 3 days) or artesunate (16 mg/kg in 7 days) according to the national guidelines.

### Passive case detection

Passive case detection of malaria was started in July 2004, and continued until the end of the study. Patients could attend either the Commune Health Centres (CHC) or consult the village health workers (VHW). The latter and the CHC health staff were trained to use rapid diagnostic tests, to take blood slides and administer the treatment to malaria patients according to the test results.*Plasmodium falciparum *cases (including mixed infections) were treated with a full course of artesunate (7 days), while *P. vivax *patients received a full course of of chloroquine (3 days); primaquine was not used. Patients attending the CHC or consulting the VHW were identified onto the census file, asked about fever in the past 48 hours, had their body (axillary) temperature registered, and a blood sample taken to detect malaria infection, i.e. a rapid diagnostic test and a thick and thin blood film for later microscopic examination. Rapid tests results were used only to decide if antimalarial treatment had to be immediately administered while microscopy on all blood films for species identification and parasite density determination was carried out later. Quality of case management, blood sampling, and reporting was assured by monthly supervision meetings with the staff of the Provincial Centre for Malariology, Parasitology and Entomology (PCMPE) and the District Health Centres (DHC), and retraining was provided whenever needed.

### Laboratory tests

#### Rapid diagnostic test

Paramax-3™ (Zephyr Biomedicals, India) rapid tests for detecting *P. falciparum*-specific histidine rich protein-2 (Pf HRP-2), *P. vivax *specific lactacte dehydrogenase (pLDH) and a pan malaria-specific pLDH were used. Four drops of buffer solution were added to the blood drop onto the sample pad on the test device and results were read after 15 minutes. A control band served to validate the test performance.

#### Microscopic examination

Thin films were fixed with methanol, then stained, together with thick films, with a 3% Giemsa solution for 45 minutes, and kept in slide boxes at room temperature. The number of asexual parasites per 200 white blood cells (WBCs) was counted and parasite densities were computed assuming a mean WBC count of 8,000/μL. A slide was defined as negative if no asexual form was found after counting 1,000 WBCs. Microscopic examination was blinded to patients' identity and location: reading and quality control was performed at the National Institute of Malariology, Parasitology and Entomology in Hanoi. Discrepant results were re-read and agreed upon by a third senior technician.

### Case definition

Patients with malaria symptoms consulting the VHW or attending the CHC were considered as suspected malaria cases. A malaria infection was defined as a positive blood slide with Plasmodium asexual forms, regardless of symptoms and parasite density. Clinical malaria was defined as a patient with fever (body temperature ≥ 37.5°C), and/or history of fever in the past 48 hours, and a positive blood slide for Plasmodium asexual forms. Recrudescence was defined as clinical malaria occurring within 28 days following the first episode. Splenomegaly was defined as any palpable spleen, independently of the Hackett classification.

### Data management and statistical analysis

Data were double entered, checked and cleaned using EpiInfo v6.04d. The data set was analysed with STATA 9.0 software (Stata Corp., College Station, TX). Descriptive statistics were used to compute malariometric indices and a survey chi-square test ("svytab" command in STATA, taking into account the cluster effect) was used to test for significant differences in proportions (p < 0.05).

The population follow up was divided in 6-month periods. Malaria incidence rates were computed by dividing the number of new cases in a given semester by the corresponding total person-semester at risk (from the census file). The latter was obtained by computing for each individual the number of days spent in the study area in relation to the actual length in days of that specific semester. Incidence rates were compared and incidence rate ratios calculated using a Poisson survey regression model ("svypoisson" in STATA, allowing for the survey design).

Tests for trend, taking into account the survey design, were performed using the "lincom" command (test for linear combination of estimates) in the survey logistic and in the survey Poisson regression models.

### Ethical considerations

The study was approved by the ethical committees of the Institute of Tropical Medicine, Antwerp, in Belgium, the National Institute of Malariology, Parasitology, and Entomology, Hanoi, as well as by the Ministry of Health in Vietnam. The fundamental principles of ethics in research on human participants were maintained throughout the study period. The research procedures were disclosed to the participants and oral informed consent was sought from them or their legal representatives. Nobody was coerced into the study and if individuals wished to withdraw, they were allowed to do so without prejudice.

## Results

### Census

Nine thousands eight hundred and seventy five individuals were registered during the census carried out in March 2004 (Table 1). The Ra-glai ethnic group represented the majority (83%) of the population, which was young (median age: 19 years), and generally with a low education level. More than half of the people were forest workers, mainly working in the forest fields, and almost all households had forest fields (96.5%, 1,801/1,867). In total, one third (33%) of the population had only daily activities in the forest, while another 25% was working and sleeping there overnight, with a substantial number of days (median 23) and nights (median 16) per month spent in the forest. ITN use in the villages was high (88.2%; a few additional people were sleeping under an untreated bed net), with a median of 2.5 people per bed net, regardless of insecticide treatment. Hammocks (locally bought, either in cotton material or knotted cord) were also popular: half of households had hammocks (48.4%). Among people staying overnight in the forest, ITN use was lower (70.4%) than in those just sleeping in the villages, hammocks were used by 16.4% of the people (with or without ITN), and 13.2% of the forest workers were sleeping without hammocks or ITNs. Socio-economic status was generally low with half (951/1,867) of the houses made of thatched bamboo, only 15% (282/1,867) made of bricks, and 46% (857/1,867) of them having no radio, TV nor motorbike.

**Table 1:** Baseline characteristics of the study population

**Study population, N = 9,875**	**n**	**%**
**Sex (ratio = 0.98)**		
- Male	4,900	49.62
**Age groups:**		
- ≤ 9 years	2,684	27.18
- 10–19 years	2,379	24.09
- 20–29 years	1,733	17.55
- 30–39 years	1,110	11.24
- 40–49 years	954	9.66
- = 50 years	1,015	10.28
**Ethnic groups:**		
- Ra-glai	8,205	83.09
- K'ho	1,339	13.56
- Kinh	314	3.18
- Others (Chu, Cham, Ede)	17	0.17
**Education level (age ≥ 20, n = 4,812):**		
- None	2,197	45.66
- Primary school	2,258	46.92
- Secondary school or higher	341	7.09
- Missing	16	0.33
**Occupation:**		
- None (children, students, retired people)	4,376	44.31
- Forest work (farming & other)	5,242	53.08
- Other (teacher, health staff.)	240	2.43
- Missing	17	0.17
**Bed net use in the village:**		
- Sleep under ITN	8,707	88.17
- Sleep under an untreated bed net	494	5
- Sleep without bed net	656	6.64
- Missing	18	0.18
**Forest activities:**		
- Never	4,176	42.29
- Only during day	3,244	32.85
- Work and sleep in the forest	2,438	24.69
- Missing	17	0.17
**Days/month spent in forest**, median [range] (n = 5,682)		23 [1;30]
**Nights/month spent in forest**, median [range] (n = 2,438)		16 [1;30]
**Bednet/hammock use in the forest (n = 2,438):**		
- Sleep under ITN	1,717	70.43
- Sleep in a hammock	319	13
- Sleep under ITN and hammock	80	3.28
- Sleep without bed-net and hammock	322	13.21

***Households N = 1,867***		
**House structure:**		
- Thatched bamboo	951	50.94
- Wooden boards	374	20.03
- Dried mud	260	13.93
- Bricks	282	15.1
**Socio economic level:**		
- No radio, TV, motorbike	857	45.9
- Only a radio	517	27.69
- Only TV	99	5.3
- TV + radio (no moto)	94	5.03
- **At least a motorbike (+/-radio, TV)**	300	16.07

### Cross sectional surveys

Malaria prevalence steadily decreased from 11.7% (178/1,518) in the first survey carried out at the end of the dry season to 4.0% (80/2,019) in the last survey carried out in December 2006, at the end of the transmission season (Table 2). This decrease was even stronger when comparing the first and last end of transmission seasons (survey 2 (S2) and survey 6 (S6)): from 13.6% to 4.0%, over 70% reduction (p < 0.001). Plasmodium falciparum and P. vivax were the most common species and decreased in similar proportions, by 66% and 70%, respectively, between S2 and S6. A few P. malariae mono-infections were identified during the first four surveys, and the prevalence of mixed infections decreased by almost 89% between S2 and S6. The parasite density was consistently higher in P. falciparum than in P. vivax infections. The percentage of gametocyte carriers also significantly decreased from 8.0% (166/2068) in survey 2 to 1.0% (21/2,019) in the last survey (p = 0.003). Most infections (86%) were detected in individuals without any sign or symptom of malaria (asymptomatic carriers) and this proportion remained stable across surveys (Table 2). Splenomegaly was uncommon, with the highest number found in the first survey (1.9%, 29/1,518), while in the other surveys the prevalence was well below 0.5%. Overall, malaria prevalence decreased with increasing age group (test for trend, p < 0.05), children less than 10 years old having the highest values across all surveys (in survey 1, children had not been included).

**Table 2:** Evolution of malariometric indices across 6 consecutive cross sectional surveys

**Cross sectional surveys: Date**	**S1** **04/2004**	**S2** **12/2004**	**S3** **04/2005**	**S4** **12/2005**	**S5** **04/2006**	**S6** **12/2006**
**Participants, N**	**1,518**	**2,068**	**2,081**	**2,089**	**2,102**	**2,018**
**Malaria prevalence: n (%)** **[95%CI]** **By species:**	178 (11.7) [6.8; 19.5]	281 (13.6) [8.8 ; 20.5]	126 (6.1) [3.5; 10.4]	173 (8.3) [4.6; 14.4]	116 (5.5) [2.6; 11.5]	80 (4.0) [2.3; 6.8]
- *P. falciparum*	85 (5.6)	148 (7.1)	57 (2.7)	103 (4.9)	61(2.9)	49(2.4)
- *P. vivax*	77 (5.1)	93 (4.5)	52 (2.5)	55 (2.6)	43 (2.0)	27 (1.3)
- *P. malariae*	3 (0.2)	2 (0.1)	2 (0.1)	7 (0.3)	0 (0.0)	0 (0.0)
- *Mixed infections*	13 (0.9)	38 (1.8)	15 (0.7)	8 (0.4)	12 (0.6)	4 (0.2)

Asymptomatic infections*	153 (86.0) [66.4; 95.0]	230 (81.9) [67.7; 90.7]	97 (77.0) [40.6; 92.4]	139 (80.3) [70.2; 87.7]	99 (85.3) [68.0; 94.1]	68 (85.0) [75.6; 91.21]

**Parasite density/μl (GM)°** **[95%CI]**						
*P. falciparum*	187.9 [129.0–273.7]	169.0 [124.1–230.1]	172.6 [112.7–264.4]	225.0 [149.1–339.7]	245.0 [154.0–389.8]	370.5 [205.8–667.0]
*P. vivax*	76.3 [59.7–97.5]	79.5 [59.8–105.9]	68.6 [47.9–98.2]	81.2 [52.1–126.5]	82.1 [52.7–127.8]	145.1 [83.5–252.4]
*P. malariae*	140.7 [54.1–365.5]	81.1 [12.7–515.8]	148.1) [39.4–557.1]	93.5 [87.8–99.7]	-	-

**Gametocyte prevalence, n (%)** **[95%CI]**	65 (4.3) [2.4; 7.4]	166 (8.0) [3.0; 19.8]	33 (1.6) [0.7 ; 3.4]	55 (2.6) [1.5 ; 4.6]	44 (2.1) [0.9 ; 4.7]	21 (1.0) [0.4 ; 2.5]

**Malaria prevalence by age group**						
- <9 years	NA	101 (17.5) [11.3; 26.0]	54 (10.4) [5.3; 19.6]	59 (11.5) [6.8; 18.8]	42 (9.1) [4.2; 18.7]	28 (6.3) [3.6; 10.8]
- 10–19 years	70 (13.6) [7.3; 23.8]	78 (15.7) [9.3; 25.3]	35 (6.6) [3.3; 12.6]	41 (7.9) [3.5; 16.6]	37 (6.9) [3.1; 14.9]	28 (5.4) [2.9; 9.8]
- 20–29 years	49 (11.8) [6.3; 20.2]	42 (10.2) [5.9; 17.1]	10 (2.4) [0.8; 7.0]	27 (6.1) [3.5; 10.5]	15 (3.3) [1.2; 9.1]	9 (2.2) [0.9 ; 4.9]
- 30–39 years	30 (11.0) [7.1; 16.7]	19 (7.5) [3.6; 15.0]	15 (5.3) [2.7; 10.0]	23 (7.7) [3.5; 16.3]	7 (2.5) [0.6; 8.9]	5 (1.8) [0.7 ; 4.9]
- 40–49 years	20 (11.1) [5.8; 20.2]	27 (13.8) [7.5; 24.2]	8 (3.9) [1.0; 14.1]	17 (9.6) [4.4; 19.8]	10 (4.4) [1.6; 11.9]	7 (3.2) [1.3; 7.8]
- ≥ 50 years	9 (6.8) [2.8; 15.6]	14 (10.5) [5.2; 20.1]	4 (3.1) [1.3; 7.5]	6 (4.3) [1.8; 10.2]	5 (3.5) [1.2; 9.8]	3 (2.0) [0.6; 6.7]

**Spleen rate**	29 (1.9) [6.8; 19.3]	7 (0.3) [8.8; 20.5]	4 (0.2) [3.5; 10.4]	5 (0.2) [4.6; 14.4]	4 (0.2) [2]	4 (0.2) [2.3; 6.8]

* % asymptomatic infections/all infections; ° GM: geometric mean

### Passive case detection

Between July 2004 and December 2006, 4,862 suspected malaria cases were identified through passive case detection (Table 3). Most of them (94.0%, 4,570/4,862) presented with fever and/or had a history of fever in the past 48 h (79.6%, 3871/4862). Malaria infection was detected in 18.4% (893/4,862) of suspected cases and P. falciparum was the dominant species (75.8%, 677/893). Plasmodium vivax represented the large majority of the remaining infections, while several mixed infections were also detected. The number of malaria cases decreased progressively with age with the highest number detected in the youngest age groups (≤ 9 years and 10–19 years old). Mean parasite density was higher for P. falciparum than for P. vivax, both in fever and non-fever cases, with higher densities among fever cases compared with non-fever cases. The 893 malaria infections were detected in 687 individuals. The large majority of patients (80.2%, 551/687) had only one malaria episode during the whole study period (Table 4). However, a few individuals had two (14.1%, 97/687) or three (4.1%, 28/687) episodes, with three individuals having experienced six malaria episodes. Twenty five recrudescences (recurrent parasitaemia within 28 days of the original episode), 21 P. falciparum, and four P. vivax patients, were detected, almost all among children under 10 (88%, 22/25).

**Figure 1:**
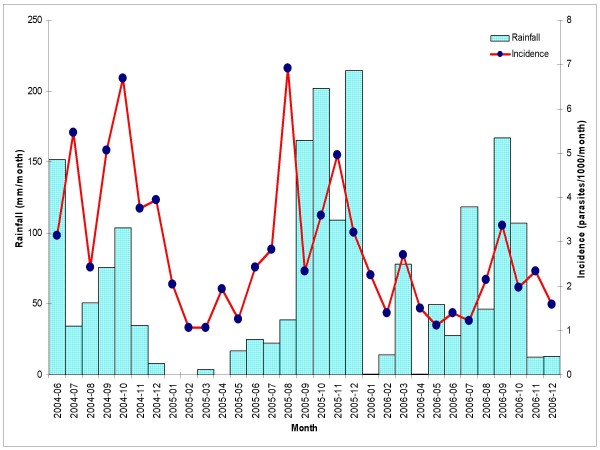
**Malaria incidence (all species) and rainfall by month during the study period**.

In the rainy season (July-December), the malaria incidence rate per semester was estimated at 25.7/1,000 person-semesters at risk in 2004, 22.4/1,000 in 2005 but only 12.7/1,000 in 2006 (Table 5): a 52% reduction within 2 years (crude IRR = 0.48; 95%CI [0.27; 0.85]; p = 0.02). Conversely, the incidence rate did not show any variation between the 2 consecutive dry seasons (Jan-Jun 2005 and 2006), though the incidence was 60% lower than that in the first rainy season (July-December 2004) (Table 5). Seasonal variations in monthly malaria incidence were loosely related with monthly rainfall (Figure 1). Malaria incidence increased with decreasing age: in each semester, children less than 10 years old experienced the highest incidence rate compared to older age groups (test for trend, p < 0.001).

**Table 3:** Characteristics of malaria patients identified by passive case detection.

**Suspected malaria cases**	**4,862**		
	***n***,	***%***	***(95%CI)***
**History of fever previous 48 hours**	3,871	79.6	(66.2 ; 93)
**Fever**	4,570	94.0	(88.9 ; 99.1)

**Malaria infection**	893	18.4	(10.4 ; 26.4)
*P. falciparum*	677	13.9	(20.5; 34.3)
*P. vivax*	164	3.4	(2.4 ; 4.4)
*P. malariae*	1	0.02	(0 ;0.06)
*Mixed*	51	1.0	(0.7; 1.6)

**Parasite density/μl (GM), without fever**			
*P. falciparum*	21	422.0	(974.0–1183.0)
*P. vivax*	8	174.0	(50.0–598.0)

**Parasite density/μl (GM°), fever cases**			
*P. falciparum*	696	1880.0	(1563.0–2262.0)
*P. vivax*	202	641.0	(477.0–863.0)

**Gametocytes carriers**	192	3.9	(2.7 ; 5.2)

**Malaria cases by age group**			
≤ 9 years	435	22.6	(13.7; 35.1)
10–19 years	202	22.2	(14.2; 32.9)
20–29 years	106	16.6	(9.5 ; 27.4)
30–39 years	74	14.1	(8.6 ; 22.5)
40–49 years	49	11.2	(7.0 ; 17.3)
≥ 50 years	27	6.3	(3.0 ; 12.5)

GM: geometric mean

**Table 4:** Number of malaria episodes per person (N = 687) over the follow-up period

**Total clinical episodes/person**	**Patients** **n (%)**
1	551 (80.2)
2	97 (14.1)
3	28 (4.1)
4	8 (1.2)
6	3 (0.4)

**Table 5:** Malaria incidence rate (per 1,000 person-semesters at risk) per semester and age group

**Age group (years)**	**Jul-Dec.04** Incidence [95%CI]	**Jan-Jun.05** Incidence [95%CI]	**Jul-Dec.05** Incidence [95%CI]	**Jan-Jun.06** Incidence [95%CI]	**Jul-Dec.06** Incidence [95%CI]
≤ 9	38.4 [18.2; 58.6]	16.9 [3.7; 30.0]	38.0 [4.7; 71.3]	20.3 [0.0; 43.4]	21.6 [0.0; 44.8]
10–19	20.7 [0.0; 42.1]	9.6 [2.2; 17.1]	22.4 [7.0; 37.7]	10.9 [0.0; 22.7]	12.9 [2.5; 23.3]
20–29	23.3 [2.6;44.0]	7.0 [1.0; 13.0]	13.7 [3.0; 24.3]	4.6 [0.0; 9.5]	9.0 [0.0; 20.1]
30–39	20.9 [2.7; 38.9]	5.9 [1.6;10.1]	20.9 [5.6; 36.3]	5.7 [0.5; 10.8]	8.1 [0.0; 16.9]
40–49	24.7 [0.0; 51.2]	5.0 [0.7;9.3]	13.9 [2.9; 25.0]	3.9 [0.0; 9.4]	8.8 [2.3; 15.2]
≥ 50	12.5 [0.0; 25.3]	2.8 [0.0; 6.2]	6.4 [0.0; 13.4]	0.9 [0.0; 3.0]	2.6 [0.0; 7.2]

**Total**	**25.7** **[7.9;43.6]**	**9.5** **[3.9; 15.1]**	**22.4** **[7.1; 37.8]**	**9.9** **[0.0; 19.7]**	**12.3** **[0.4; 24.2]**

## Discussion

Ninh Thuan has always been considered one of the most endemic malaria provinces in Vietnam [6,7]. Indeed, the first survey, carried out in April 2004, at the end of the low transmission season, showed a relatively (for Vietnam) high value for malaria prevalence (11.7%). The large proportion of asymptomatic infections seems to indicate that transmission is sufficiently intense to stimulate some partial immunity able to control malaria parasitaemia and prevent its evolution towards clinical disease. Indeed, the highest incidence of clinical attacks observed among children <9 years of age and its decreasing value as age increases supports this hypothesis, i.e. people living in the study area are repeatedly exposed since infancy to malaria infection and acquire some partial immunity. This is different from a previous study carried out in the neighbouring province of Binh Thuan, where the malaria prevalence was 5% or less and where children were not particularly at risk [11]. The high malaria prevalence in small children in Ninh Thuan seems to indicate that transmission occurs in the villages themselves. Indeed, in Ninh Thuan the villages in the study area are located close or within the forest, increasing the probability of contact with the main vector *Anopheles dirus s.s*., asylvatic species that is highly anthropophilic. In contrast, in Binh Thuan the study village was located along the district road and relatively far from the deep forest so that the malaria risk was particularly high in individuals with forest activities but not in small children [11]. The more intense transmission in Ninh Thuan as compared to Bin Thuan is supported also by the large proportion of asymptomatic cases detected by cross-sectional surveys in the former. A similar situation has been described in neighbouring countries, such as Cambodia [13], Indonesia [14] or Burma [15], where the malaria prevalence was comparable to that found in Ninh Thuan and with children having a higher risk of malaria infection. Obviously, the large proportion of asymptomatic malaria carriers in Ninh Thuan maintains malaria transmission in this area and represents an obstacle to malaria control. It is unclear what might be the impact of these asymptomatic infections on the carrier's health; the haematological status of the survey participants was not determined, mainly because in the study carried out in Binh Thuan no case of anaemia was detected [11]. However, asymptomatic infections were much less common. In Africa, asymptomatic infections have been associated with a higher risk of anaemia and lower school performances [16-18].

Within a relatively short period, both the prevalence of malaria infection and the incidence of clinical cases decreased substantially, more than 70% and 50%, respectively. Prevalence decreased steadily over the 2.5-year follow up, while for the incidence such a decrease occurred only during the last 6-month period as the incidence peak usually observed during the second part of the year did not occur. It is difficult to attribute the observed trend to a specific cause; however this is unlikely to be a natural trend in the study area since the malaria incidence in the two neighbouring provinces of the study area, i.e. Lam Dong and Khan Hoa province, experienced much smaller reductions in malaria incidence, i.e. 13% and 22%, respectively. These provinces had low incidence rates in 2004 (between 0.6 and 1.3/1,000 population). In other provinces from Central Highlands such as Dak Lak, with an annual incidence rate of 4.5/1,000 in 2004, the reduction by 2006 was only by 5% [6,7] Therefore, considering that the environment did not change dramatically and that the yearly rainfalls did not vary considerably during the whole study period, the observed decrease may reflect the impact of the malaria surveillance system set up for the study, i.e. the prompt availability of diagnosis and treatment at village level. In this area, trained VHWs could use rapid diagnostic tests and treat the confirmed cases of *P. falciparum *or *P. vivax *(as well as other species suspected cases) with either artesunate or chloroquine, respectively. VHW are often employed by health programmes in rural and remote areas where public health services are not always or easily accessible and available. In Vietnam, 84% of villages (61,664/73,462) have VHWs who have participated to malaria control activities, usually health promotion and in some areas also rapid diagnosis and prompt treatment [3]. In the present study, about half of the confirmed malaria cases by PCD were detected by the VHWs in their respective villages. Therefore, the most important contributing factor on the observed decreasing trend in the study area may be the activity of the VHWs. Our results confirm previous reports about VHWs having played an important role in malaria diagnosis and treatment in many different settings and for more than 45 years [19]. Malaria morbidity and mortality were significantly reduced when the rural population at risk for malaria were treated at village level [20]. In Cambodia, after the scaling up of the VHW project (rapid tests + pre-packaged combination therapy for malaria) in Rattanakiri province, the annual malaria incidence decreased by more than 60%, from 165/1,000 in 2006 to 64/1,000 in 2007 [21]. Similarly, in a remote area of Mindanao, the Philippines, the parasite prevalence was significantly lower among individuals living in villages with a resident VHW [22].

In conclusion, despite a successful control program, malaria transmission in Vietnam is still ongoing at a relatively high intensity in some remote areas. Nevertheless, in Ninh Thuan province, a significant decreasing trend in malaria prevalence and incidence has been observed in a short period (2.5 years). This is probably due to the set up of a passive case detection system based on VHW, indicating that a major impact on the malaria burden can be obtained whenever prompt diagnosis and adequate treatment are available.

## Competing interests

The authors declare that they have no competing interests.

## Authors' contributions

TND contributed to the study design, study coordination and supervision, field work, data entry, cleaning and analysis, and paper writing. AE contributed to the study design, study coordination and supervision, field work, statistical analysis and reviewed the manuscript. HLX contributed to the study design, study coordination and supervision, and reviewed the manuscript. TLK contributed to the study design and reviewed the manuscript. TNN contributed to the field work, data entry & cleaning, the study coordination and supervision. XNX contributed to the field work, data entry & cleaning, the study coordination and supervision. KPV contributed to the field work, data entry & cleaning, the study coordination and supervision. MC reviewed the paper. UD contributed to the study design, study coordination and supervision, data analysis and reviewed the manuscript.
